# Effect of Alternate Nostril Breathing Exercise on Experimentally Induced Anxiety in Healthy Volunteers Using the Simulated Public Speaking Model: A Randomized Controlled Pilot Study

**DOI:** 10.1155/2017/2450670

**Published:** 2017-10-11

**Authors:** Ashwin Kamath, Rathnakar P. Urval, Ashok K. Shenoy

**Affiliations:** ^1^Department of Pharmacology, Kasturba Medical College, Manipal University, Mangaluru, Karnataka, India; ^2^Department of Pharmacology, Kanachur Institute of Medical Sciences, Mangaluru, Karnataka, India

## Abstract

A randomized controlled pilot study was carried out to determine the effect of a 15-minute practice of ANB exercise on experimentally induced anxiety using the simulated public speaking model in yoga-naïve healthy young adults. Thirty consenting medical students were equally divided into test and control groups. The test group performed alternate nostril breathing exercise for 15 minutes, while the control group sat in a quiet room before participating in the simulated public speaking test (SPST). Visual Analog Mood Scale and Self-Statements during Public Speaking scale were used to measure the mood state at different phases of the SPST. The psychometric scores of both groups were comparable at baseline. Repeated-measures ANOVA showed a significant effect of phase (*p* < 0.05), but group and gender did not have statistically significant influence on the mean anxiety scores. However, the test group showed a trend towards lower mean scores for the anxiety factor when compared with the control group. Considering the limitations of this pilot study and the trend seen towards lower anxiety in the test group, alternate nostril breathing may have potential anxiolytic effect in acute stressful situations. A study with larger sample size is therefore warranted. This trial is registered with CTRI/2014/03/004460.

## 1. Introduction

Anxiety is a normal human emotion that serves to alert and enable the person to deal with perceived stressful situation. The anticipatory and adaptive responses stimulated by anxiety when out of proportion to the stressful situation can result in significant psychological and social impairment [1]. Disproportionate anxiety or that occurring in the absence of a stressor can be controlled by pharmacotherapy. However, anxiolytics are associated with problems of habit formation, withdrawal effects, overdosage, or other undesirable effects [2]. A number of randomized and nonrandomized controlled trials have tested yoga as an intervention with respect to anxiety and anxiety disorders [3]. Yoga, a commonly practiced mind-body intervention, involves a combination of physical activity, breathing exercise, and meditation. It is purported to be potentially useful in a number of physical and mental illnesses [4]. However, owing to the diversity of conditions treated and methodological issues in many studies, the evidence to support the effectiveness of yoga in treating anxiety or anxiety disorders, in general, requires further research [3].

Among the various yoga practices, the alternate nostril breathing (ANB) is a fairly simple and commonly performed exercise. The practice of ANB is traditionally considered to relieve mental unrest and promote physical and mental balance [5, 6]. There have been studies to assess the effects of ANB technique on specific physiological and cognitive functions [7]. There is evidence for a balancing effect of ANB on the activity of both cerebral hemispheres [8]. Also, the practice of ANB has been shown to improve visuospatial memory and improve performance in letter cancellation task [9]. A more recent study also showed that ANB exercise produced better cancellation task scores as measured by the effect on P300 auditory event-related potential. Letter cancellation task requires selective attention and concentration [10]. Since anxiety is associated with a general inability to maintain attentional focus, the findings of these studies provide evidence for a potential anxiolytic effect of ANB [7].

Yogic breathing exercises are also known to produce acute changes in the cardiovascular parameters [11]. In particular, ANB has been shown to reduce blood pressure and improve heart rate variability measures by a shift in the autonomic tone towards vagal dominance [12–14]. These findings provide supportive evidence for the potential antianxiety effects of ANB considering the strong correlation between anxiety states, sympathetic overactivity, and attendant changes in heart rate and blood pressure.

The anxiolytic potential of drugs has been tested in human experimental models using psychological means. Environmental stimuli or contexts have been used in healthy volunteers to induce anxiety under ethical conditions [15]. Although the level of anxiety induced is low, such studies have yielded valuable results. Public speaking anxiety is highly prevalent in the general population, particularly among students. This is the basis for the clinical anxiety model, simulated public speaking, which consists of speaking in front of a video camera [15]. It is presumed that social anxiety results from negative self-perception or perceived negative evaluation by other people in social situations [16]. Various scales such as the Visual Analog Mood Scale (VAMS) developed by Norris and the Self-Statements during Public Speaking (SSPS) scale can be used to quantify the anxiety [16–18]. The simulated public speaking test (SPST) has been shown to provoke anxiety in healthy volunteers irrespective of the trait level anxiety [15].

Considering the potential anxiolytic effects of ANB and its acute effects on the cardiovascular parameters, we hypothesized that a short practice of ANB exercise also might influence the mood states and thereby modify anxiety levels. Hence, the objective of our study was to determine the effect of a short practice of alternate nostril breathing exercise on anxiety induced by the SPST in healthy young adults.

## 2. Method

**Figure 1:**
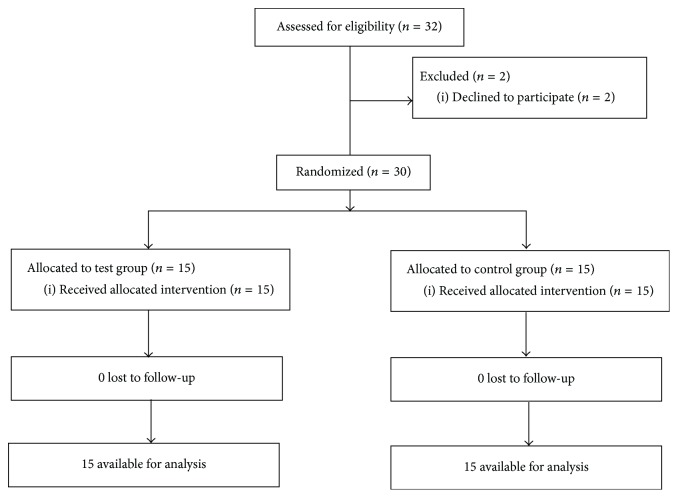
CONSORT diagram.

The study was initiated following approval from the institutional ethics committee and conducted in compliance with the principles of the Declaration of Helsinki. The study protocol has been registered in the Clinical Trial Registry of India (CTRI/2014/03/004460). The study participants consisted of second-year undergraduate medical students. A batch of 124 students was provided preliminary information about a study involving performing a breathing exercise and evaluation of anxiety and invited to attend a session on study details and informed consent. Thirty-two students reported to the study facility on the scheduled date and time (Figure 1). Two students dropped out before the informed consent session for personal reasons. The remaining 30 students provided written informed consent. The students were provided the study details. However, they were told that the method used to induce anxiety would be revealed only after baseline measurements. They were also informed that they were free to withdraw from the study if they were uncomfortable with the study procedure or for any other reason.

The inclusion criteria for participation in the study were healthy and yoga-naïve participants of either gender between 19 and 24 years of age. The exclusion criteria were history of any acute or chronic illness at the time of participation and history of intake of any medication within a week prior to participation in the study. The 30 participants were equally divided into the test and control groups by simple random allocation using a computer-generated random number table. The test group performed the ANB exercise for 15 minutes following baseline measurements, while the subjects in the control group were made to sit comfortably in a quiet room for an equal duration. This was followed by the simulated public speaking test.

### 2.1. Alternate Nostril Breathing [4, 10]

The instructions for the practice of ANB and demonstration were provided by the study's author (Ashwin Kamath) to the subjects followed by a practice session of ANB and clarification of any doubts regarding the procedure before the 15-minute intervention period began. The subjects were instructed to sit comfortably on a chair with the spine erect. We did not adopt the traditional cross-legged posture in order to avoid any possible discomfort in the yoga-naïve participants. The exercise began with exhalation through both nostrils, followed by closing the right nostril with the thumb of right hand. The subjects then inhaled slowly through the left nostril. After complete inhalation, the left nostril was closed with the little and ring fingers of right hand followed by the opening of the right nostril and exhaling through it. The subjects then inhaled through the right nostril followed by exhalation through the left nostril. This formed one round of ANB. This was continued for 15 minutes. The subjects were instructed to close their eyes and focus their attention on the breath throughout the exercise.

### 2.2. Simulated Public Speaking [16, 19]

The study participants were asked to prepare a short speech on a topic (advice to the Prime Minister regarding controlling environmental pollution) following the ANB exercise in the test group and resting period in the control group. The topic was uniform for all participants and was provided on the spot by the investigator. Two minutes were given to prepare a 4-minute speech. Participants were made to deliver the speech facing the in-built camera of a laptop such that they could see their face on the laptop screen. The participants were told that the speech would be recorded on the laptop and would be evaluated by a psychologist.

### 2.3. Study Measurements

Two psychometric measures were used to determine the mood state of the study participants: VAMS and SSPS scale. The VAMS developed by Norris is a 16-item scale to measure state-level anxiety and other subjective states [17]. The study participant has to mark a point on a horizontal line between two adjectives of opposite meaning which represents his/her present feelings. The 16 items are grouped into four factors: anxiety, sedation, cognitive impairment, and discomfort. The items calm–excited, relaxed–tense, and tranquil–troubled comprise the anxiety factor. Two words in the 16-item scale, “gregarious” and “amicable,” were replaced with the words “sociable” and “friendly,” respectively, for better comprehension by the study participants [18]. The SSPS aims to measure self-perception of performance in the specific situation of public speaking [16]. The scale is comprised of 10 items, rated on a Likert scale from 0 (strongly disagree) to 5 (strongly agree), which are organized into two subscales of five items each, for positive (SSPS-P) and negative (SSPS-N) self-evaluation. SSPS-N, in particular, can differentiate anxious from nonanxious individuals, with those scoring higher being more anxious [16].

The sequence of events is presented in Table 1.

### 2.4. Statistical Analysis

Gender distribution between the experimental groups was compared using Fisher's exact test. The scores for the four VAMS factors and SSPS were transformed by calculating the difference between the scores during each phase and the baseline score for the same subject. These delta scores were subjected to repeated-measures analysis of variance (ANOVA). The analyzed factors were phases (five phases: baseline, prestress, anticipatory phase, speech performance, and poststress phase) and groups (test and control). Within-subjects factor was phase and between-subjects factor was group. Repeated-measures ANOVA was performed for each variable (VAMS: anxiety, sedation, cognitive impairment, and discomfort; SSPS-P and SSPS-N). Wherever the sphericity assumption was violated, the degrees of freedom were corrected using the Greenhouse-Geisser epsilon. Baseline VAMS and SSPS scores were compared using Student's *t*-test. Data analysis was performed using Statistical Package for Social Sciences, IBM Corporation, Version 11.5. A *p* value < 0.05 was considered statistically significant.

## 3. Results

### 3.1. Gender Distribution and Baseline Scores

40% (6/15) of the subjects in the test group and 20% (3/15) in the control group were males (*p* = 0.427). There was no significant difference between the experimental groups in the mean scores for the four VAMS factors and SSPS at baseline (Table 2).

### 3.2. Psychometric Measures

**Figure 2:**
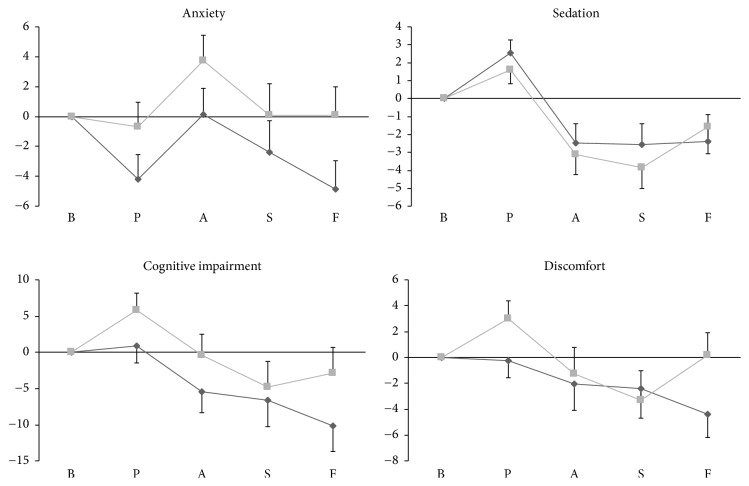
Comparison of the delta scores of Visual Analog Mood Scale (VAMS) factors in the test and control groups during various phases of the simulated public speaking test. The delta scores were obtained by calculating the difference between the VAMS score for each phase and the baseline score. The dark-gray line represents the test group, and the light-gray line represents the control group. The horizontal axis represents the various experimental phases, and the vertical axis represents the VAMS factor scores. The vertical bars represent the standard error. B, baseline; P, prestress; A, anticipatory phase; S, speech performance; F, poststress.

The changes in relation to the baseline measures of VAMS factors in the study groups are shown in Figure 2.

#### 3.2.1. Anxiety Factor

Repeated-measures ANOVA showed a significant effect of phase (*F*_4, 112_ = 5.283; *p* = 0.001) but not group or phase by group interaction. The anxiety scores (mean ± standard deviation [SD]) for the test and the control groups during the prestress, anticipatory, speech performance, and poststress phases were 9.96 ± 4.98 versus 13.81 ± 7.69, 14.32 ± 6.28 versus 18.22 ± 7.05, 11.78 ± 7.63 versus 14.58 ± 8.75, and 9.30 ± 6.85 versus 14.61 ± 7.75, respectively.

#### 3.2.2. Sedation Factor

Repeated-measures ANOVA showed a significant effect of phase (*F*_2.23, 62.54_ = 16.14; *p* < 0.001) but not group or phase by group interaction. The largest difference between the test and control groups' scores (mean ± SD) was seen in the speech performance phase (5.00 ± 4.60 versus 6.20 ± 4.29, resp.).

#### 3.2.3. Cognitive Impairment Factor

Repeated-measures ANOVA showed a significant effect of phase (*F*_2.34, 65.73_ = 6.562; *p* = 0.002) but not group or phase by group interaction. The largest difference between the test and control groups' scores (mean ± SD) was seen in the poststress phase (18.56 ± 15.27 versus 28.64 ± 12.26, resp.).

#### 3.2.4. Discomfort Factor

Repeated-measures ANOVA showed a significant effect of phase (*F*_2.26, 63.39_ = 3.527; *p* = 0.03) but not group or phase by group interaction. The largest difference between the test and control groups' scores (mean ± SD) was seen in the poststress phase (10.00 ± 8.19 versus 17.16 ± 5.06, resp.).

**Figure 3:**
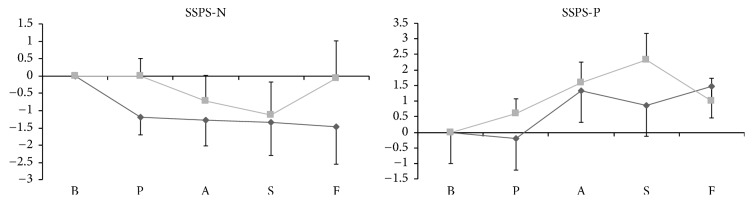
Comparison of the delta scores of Self-Statements during Public Speaking (SSPS) scale in the test and control groups during various phases of the simulated public speaking test. The delta scores were obtained by calculating the difference between the SSPS score for each phase and the baseline score. The dark-gray line represents the test group, and the light-gray line represents the control group. The horizontal axis represents the various experimental phases, and the vertical axis represents the SSPS scores. The vertical bars represent the standard error. B, baseline; P, prestress; A, anticipatory phase; S, speech performance; F, poststress.

The scores of SSPS-P and SSPS-N did not differ significantly between the groups at baseline. Repeated-measures ANOVA showed no significant effect of phases or groups for SSPS-N (Figure 3). However, a significant phase effect was seen for SSPS-P (*F*_2.77, 77.53_ = 4.564; *p* = 0.007).

## 4. Discussion

We studied the effect of alternate nostril breathing on experimentally induced anxiety in yoga-naïve healthy subjects using the simulated public speaking model. Two psychometric measures were used to evaluate the effect of the intervention: Visual Analog Mood Scale and Self-Statements during Public Speaking scale. The results of the study show that there was a significant phase effect on the VAMS scores, suggesting that the simulated public speaking model did induce significant anxiety in the study subjects. However, the short practice of ANB had no statistically significant effect on anxiety levels, as measured using the psychometric measures, compared to the control group. A trend towards lower anxiety scores was seen in the test group. No phase or group effect was seen with SSPS-N, which is more indicative of anxiety states, although a phase effect was present for SSPS-P.

A few factors need to be considered in the interpretation of the results of our study. We studied the acute effects of a short practice of ANB on anxiety in yoga-naïve individuals. The purported neurophysiological effects of ANB might require longer practice periods to manifest [20]. Hence, the results obtained with our study participants might not be representative of the effects seen in well-trained individuals. Earlier studies have shown acute changes in the autonomic tone and cardiovascular activity following a short practice of ANB [11–14]. However, the present study differed from these in terms of the time interval between the cessation of ANB exercise and measurement of its effect on induced anxiety. There was a time gap of 14 minutes between the cessation of ANB exercise and measurement of VAMS and SSPS in the anticipatory phase (Table 1), during which the subjects were provided instructions about SPST and allowed to prepare for the speech. This delay may have resulted in a waning of the anxiolytic effect of ANB. We also looked for any possible effect of gender on the study results by factoring it in the repeated-measures ANOVA. No difference in the study results was seen.

The magnitude of anxiety generated by the simulated public speaking model in healthy volunteers needs to be taken into account. Simulated public speaking may not induce marked changes in the physiological measures of anxiety as compared to the real-world stressful situation [21]. Hence, although the simulated public speaking model does induce measurable anxiety in healthy volunteers, it might not be large enough to observe a significant difference in a small sample. It should be noted that SPST has been successfully used in the evaluation of anxiolytic and anxiogenic drugs using subjects who showed significant anxiety traits at baseline. Since we intended to study the effects of ANB in otherwise normal individuals irrespective of the baseline anxiety traits, the magnitude of the intervention effect might have been smaller. However, the results of the present study did show a trend of the scores being lower in the test group in comparison with those of the control group.

Also to be considered is the appropriateness of use of SPST coupled with the psychometric measures for determining the effects of a short practice of ANB. To the best of the authors' knowledge, this is the first study to determine the effect of yoga intervention on anxiety induced by the simulated public speaking model. It has been hypothesized that SPST induces two types of emotional states [15]. The anticipatory anxiety is akin to the conditioned anxiety generated by aversive conditioning and is responsive to benzodiazepines. The performance anxiety is related to serotonin and is increased or decreased by drugs modulating the serotonergic system. Correlation of improvement in mood and decrease in anxiety to increases in thalamic GABA levels following yoga intervention has been reported in a study [20]. The increase in GABA levels is probably secondary to increase in vagal activity. Whether yoga has any significant effect on the brain's serotonin levels has been less well studied, although it is a common alternative therapy in depression [22]. Considering the fact that there was a difference between the two groups in the anxiety measures following the equal baseline readings with a uniform trend of lower anxiety in the test group, we are justified in using the current methodology.

Our study has limitations. The sample size was small, and hence the study may have been unable to detect a small but significant intervention effect. The yoga-naïve participants performed the ANB exercise following only a short practice session, which may have affected the quality of performance. We did not include the objective measures of anxiety such as variation in heart rate and blood pressure, which would have yielded a more comprehensive result along with the psychometric measures. A study with a larger sample size involving participants with prior training in the breathing exercise and inclusion of objective physiological measures might provide more conclusive results.

## 5. Conclusion

A 15-minute practice of ANB exercise did not produce a significant decrease in the anxiety induced by simulated public speaking in yoga-naïve subjects. However, the test group had lower VAMS anxiety scores compared with those of the control group, suggesting a potential anxiolytic effect of ANB exercise in acute stressful situations. Considering the limitations of the current pilot study and the trend seen towards lower anxiety scores in the test group, a study with a larger sample size to determine the potential acute anxiolytic effects of ANB exercise is warranted.

## Figures and Tables

**Table 1:** Time sequence of study-related procedures.

Time (minutes)	Phase	Procedure
−0:30		Instructions about the intervention and measurements
−0:15	Baseline (B)	VAMS, SSPS
0:00	Intervention	ANB or control
0:15	Prestress (P)	VAMS, SSPS
0:25		Instructions about the simulated public speaking test
0:27		Speech preparation
0:29	Anticipatory phase (A)	VAMS, SSPS
0:35		Start of speech
0:37	Speech performance (S)	VAMS, SSPS
0:43		Continuation of speech
0:45		End of speech
0:55	Poststress (F)	VAMS, SSPS

VAMS, Visual Analog Mood Scale; SSPS, Self-Statements during Public Speaking; ANB, alternate nostril breathing.

**Table 2:** Baseline VAMS and SSPS scores in the test (ANB) and control groups.

Baseline parameter	Test (*N* = 15)	Control (*N* = 15)	*p* value
	Mean ± SD	Mean ± SD	
VAMS-anxiety	14.17 ± 4.78	14.51 ± 5.08	0.852
VAMS-sedation	7.57 ± 3.73	10.06 ± 4.17	0.096
VAMS-cognitive impairment	28.72 ± 7.92	31.52 ± 9.52	0.389
VAMS-discomfort	14.41 ± 6.73	17.01 ± 7.72	0.335
SSPS-P	18.93 ± 3.03	17.13 ± 3.07	0.117
SSPS-N	14.60 ± 2.72	14.67 ± 3.39	0.953

VAMS, Visual Analog Mood Scale; SSPS, Self-Statements during Public Speaking; SSPS-P, positive statements; SSPS-N, negative statements; ANB, alternate nostril breathing.
